# Public knowledge about oral cancer in Uganda: a free dental camp experience

**DOI:** 10.1108/jhr-07-2018-0062

**Published:** 2019-07-07

**Authors:** Rose Chalo Nabirye, Adriane Kamulegeya

**Affiliations:** Department of Nursing, Makerere University, Kampala, Uganda; Department of Dentistry, Makerere University, Kampala, Uganda

**Keywords:** Uganda, Cancer awareness, Cancer knowledge, Cancer risk factors, Oral cancer, Public cancer knowledge

## Abstract

**Purpose–:**

The purpose of this paper is to assess the levels of awareness and knowledge about oral cancer, its causes and or risk factors among Ugandan patients seeking oral healthcare.

**Design/methodology/approach–:**

This was a cross-sectional study on adult patients who attended a free dental camp. An assistant-administered questionnaire either in English or Luganda was provided to every even-numbered registered adult who consented to participate in the study. Information on demographics and known risk factors for oral cancer were captured. The two knowledge questions on oral cancers were scored by adding up all the correctly identified causes, non-causes and risk factors then scored out of the total. Data analysis was done by calculating proportions, Student’s’ *t*-tests and *χ*^2^ tests with significant *p*-value set at 0.05.

**Findings–:**

The results showed a low level of awareness/knowledge about oral cancer in studied population. In total, 60 percent and less than 50 percent of respondents identified smoking and alcohol use as risk factors for oral cancer, respectively. Majority of respondents (88.8 percent) would seek help from medical personnel if diagnosed with oral cancer. Screening for cancer was low despite awareness and knowledge that it improves the chances of successful treatment.

**Research limitations/implications–:**

Emphasis on risk factors including alcohol use in public health messages, use of mass media, religious and community leaders to disseminate messages to the communities and further research were recommended.

**Practical implications–:**

We need to emphasize the role of alcohol in oral cancer causation just as we do for tobacco consumption.

**Originality/value–:**

No study has been conducted in Uganda on the level of awareness yet the incidence of the disease and use of high-risk products are rising.

## Introduction

Despite the fact that oral cancer is a rare malignancy, its effect and treatment sequela may lower the affected individual’s quality of life to varying degrees dependent on the affected site and treatment approach[1]. Globally, oral cancer ranks 12th in incidence with records of male incidence at about three times that of females[2]. Although advances in treatment have resulted in tremendous improvements in prognosis and survival in high gross domestic product (GDP) countries, this is yet to be seen amongst the poorer counterparts. Additionally, the five-year survival is still just around 50 percent and worse in the low GDP countries compared to their high GDP counterparts[3]. Furthermore, glaring differences in survival have been reported on the basis of ethnicity, income levels and developed vs developing countries[4, 5]. Furthermore, even though reports from the Kyadodndo Cancer Registry, the oldest established cancer registry in Africa, do not capture oral cancer[6], it is worth noting that the registry covers a fraction of the country whose population has changed over the years. In addition, Ugandan patients with head and neck cancers have been shown to report their condition mostly at the advanced stages of the disease which disadvantages their outcomes[7].

Sub-Saharan African countries are mainly in the category of low GDP nations and unfortunately have been noted to bear the brunt of higher incidences of cancers coupled with the worst survival compared to developed nations[5, 8]. To make matters worse, researchers found that adopting increased western and urban lifestyles characterized by an increase in alcohol and tobacco use provided a fertile ground for the increase in cancer incidence. Since tobacco use and alcohol intake are known oral cancer-causing agents, researchers are likely to continuously see a rise in the incidence of the disease unless preventive interventions are instituted[9].

Additionally, human life expectancy has seen a dramatic rise, yet it is a known fact that longer life spans come with an increased risk of developing cancer with advancing age[10]. Therefore, researchers are likely to see an increase in cancer cases for some time to come.

Uganda is ranked high in global alcohol consumption and it is catching up on tobacco product use. This is worsened by the increased availability of cheap sachet liquor and tobacco products such as cigarettes, shisha and others that have become more readily importable with globalization and free market economy benefits[11–13]. Additionally, the increased use of chewed herbs such as khat and other imported products whose contents may have carcinogenic substances are on the increase. To make matters worse, Uganda’s cancer control program at the moment is concentrating on cervical, prostate and breast cancer and excluding other cancers[14]. This is likely to see a rise in the incidence of oral cancer since it has been shown to be on the increase in countries with no preventive strategies[15]. Although Uganda and many African countries have passed tobacco control legislation, they are struggling to have the same legislation for alcohol. Additionally, the implementation of tobacco control legislation is still wanting. Given the known association between smoking and alcohol intake on the risk of acquiring oral cancer[16], Uganda is poised to see a spike in this disease unless preventive measures are taken more seriously.

In recent reports, the human papillomavirus (HPV) has been shown to be a major causative factor in certain oral and oropharyngeal carcinoma affecting mainly young age groups with the other known risk factor attributed to oral sex[17, 18]. Fortunately, Sub-Saharan African countries including Uganda have initiated HPV vaccination for cervical cancer, targeting young females before sexual exposure and hopefully protecting them against HPV oral/oropharyngeal cancers as well. However, this still leaves a good section of the population who may be predisposed to HPV-related cancers. Given the country’s high HIV prevalence[19] plus the known immune-modifying effect of HIV/AIDs resulting in the persistence of HPV[20], we may see a rise in oral and oropharyngeal cancers for the foreseeable future. Highly active antiretroviral therapy has seen an increment in the life expectancy of these patients putting them in the high-risk group of cancer as they become elderly.

There are a number of other real and perceived risk factors for oral cancer. These range from proven ones such as sun exposure[21] to controversial ones that include the likes of khat[22], marijuana[23] and fruit consumption[24]. Their effect in our setting is hard to gauge due to higher melanin levels, legislation against some of the agents and difficulty in ascertaining fruit and vegetable consumption.

As oral health service providers interact with patients, they have a unique opportunity to address known risk factors. Additionally, as we develop oral health education we can incorporate oral cancer public health messages. Thus, researchers must take our place in preventive and early diagnosis of oral cancers[25]. As oral health care providers, researchers are limited in their ability to do much in averting an increase in oral cancer incidence and mortality by participating in general population and patient education with disease-specific prevention and early signs information[25]. At the moment, researchers do not know the level of public knowledge when it comes to oral cancer in the country. This study’s main objective was to find out the level of awareness about oral cancer and its causes among Ugandan patients seeking oral health care services. This study will form the basis of a plan to implement appropriate public health messages targeting oral cancer.

## Methods

A cross-sectional study was conducted on adult patients who attended a widely advertised free dental treatment camp at Mulago sponsored by the National Social Security fund and the Rotary Club of Kampala North. A random selection between taking odd-or even-numbered adult patients who attended was conducted during June 2016. The raffle fell on the even-numbered attendees and as such, the researchers recruited every even-numbered registered adult and if he/she declined, we then took the next even one. All consenting even-numbered adult patients who attended this camp were included as long as they were comfortable with speaking either English or Luganda. The researchers excluded all patients who could not speak either of the two languages.

An assistant administered the questionnaire either in English or Luganda. The assistants who were fifth-year dental students were chosen based on their fluency in both languages. The researchers trained and discussed the translated questionnaire with them. Then, the questionnaires were pretested on ten third-year dental students who got the Luganda version first. A joint debriefing session was held with the authors and both groups of students. In this session, the researchers agreed on the translations to be used for the final Luganda questionnaire.

The questionnaires captured information including demographics, details on the use of alcohol and smoking, oral sex practice, cancers known to the patient, family history of cancer, causes plus risk factors for oral cancer, experience with oral cancer screening and some general knowledge questions about cancer. The knowledge questions intentionally had both right and wrong options in an effort to reduce the effect of guesswork. The incorrect options included items such as dental fillings and hot chillies causing cancer. During data entry and cleaning, the two knowledge questions on oral cancers were scored by adding up all the correctly identified causes, non-causes and risk factors that were then scored out of the total options.

The researcher also had some attitudinal questions that utilized a Likert scale to try and capture the complexities of aspects such as the perceived role of fatalism and witchcraft in cancer causation.

This study was approved by the Mulago Hospital Research and Ethics Committee as an expedited review on June 20, 2016 (MREC 1211).

### Sample size calculation and statistical analysis

The sample size was calculated based on a study from Iran that assessed knowledge about oral cancer among dental patients at 11.6 percent[26]. Using a 95% confidence interval and width of 0.1, with the help of sample size calculators for clinical research from the UCSF clinical and translational research institute, a minimum sample size was 167.

SPSS 15 was used to perform simple proportion tests, Students’ *t*-tests and *χ*^2^ tests with *p* set at 0.05. The researchers also calculated the power with which we can draw conclusions with our sample size as compared with the Iranian study[26].

## Results

There were 522 adult patients who attended the camp out of a total of 1,089. Of these, given our selection based on every even-numbered adult patient, the researchers had a sample size of 261. Of these, 188 accepted the invitation to participate and filled the questionnaires, representing 72.1 percent of the target population.

The female participants were 56.8 percent, and 43.2 percent were male. The age range was 18–90 with a mean age of 34.4 ± 15.5 years. Compared to the Iranian study[26], the researchers had a power of 80 percent to detect *P*_1_ at 96 percent given our sample size.

### Awareness about oral cancer

Up to 93.0 percent claimed that they had never heard of oral cancer. This was the worst level of awareness of all the cancers the researchers inquired about including lung, skin, cervical, prostate, liver, throat and colon cancers at 34.6, 40.0, 35.7, 58.7, 67.0, 50.3 and 54.6 percent, respectively, with pelvic cancer pointed out as potential cancer by up to 22.0 percent. The higher income group was more aware of the existence of oral cancer as shown in Table I.

There was no statistically significant difference between the mean percentage knowledge question scores between male and females for 50.9±13.5 percent and 51.5±12.3 percent, respectively. Likewise, there was no statistically significant difference in the knowledge levels between the different occupations, income groups and age groups at *p* 0.712, 0.875 and 0.264, respectively. Neither the death of a family member due to cancer nor the feeling of being at risk of oral cancer had an effect on awareness of oral cancer at *χ*^2^ =.845; p 0.959 and *χ*^2^ = 1.002; *p* 0.606, respectively.

### Awareness/knowledge about risk factors of oral cancer

Most of our respondents correctly identified smoking (66.5 percent) as a risk factor for oral cancer while alcohol (44.9 percent), oral sex (34.1 percent), sun exposure (11.4 percent) and lack of fresh fruits in the diet (11.4 percent) were identified as other risk factors. Age above 50 years and HIV were also reported as possible risks that increase a person’s chances of getting oral cancer (23.2 and 35.7 percent), respectively. On the other hand, marijuana was identified by 33.0 percent, khat (25.4 percent), car fumes (21.6 percent), chilli/hot pepper (8.6 percent) and dental fillings (13.0 percent) were also identified. However, people who were 49 years and older chose to live with HIV more frequently as one of the risk factors for oral cancer as shown in Table I. Additionally, respondents who had a relative dying from cancer identified tobacco use and living with HIV as risk factors for oral cancer more often than those who did not. Likewise, those who had a relative who passed on due to cancer pointed out tobacco as a risk factor for oral cancer more often compared to those who did not.

Only 6.5 percent ever had a cancer examination while 3.2 percent were not sure. In total, 33 percent reported a relative who died from cancer while 17.3 percent did not know if any of their family members had ever died of the disease.

There were several questions that had a Likert scale on factors that determine how well patients with cancer do and the results are shown in Table II. Close to 19 percent felt they were at the risk of oral cancer.

In the case of a cancer diagnosis, the respondents gave several sources of help in managing the disease. These included: medical personnel (88.1 percent), balanced diet (31.4 percent), prayers (28.1 percent) use of food supplements (19.5 percent), pastors (11.9 percent) and traditional healers (3.8 percent).

### Alcohol use

In total, 57 percent of respondents reported never having had any alcoholic drinks; however, of those who did, 31.9 percent reported taking it on a daily basis while an equal number said they do so weekly. Beer was the most consumed alcohol at 63.2 percent while hard liquor was at 15.8 percent with 14.5 percent taking both beer and hard liquor. It was hard to determine the amounts and strength of liquor taken. This is due to the fact that beers come in different volumes and concentrations while the local liquor is yet to be standardized countrywide. Interestingly, people who had consumed alcohol pointed out tobacco use and age above 50 years as risk factors for oral cancer compared to those who had not. However, they did not point out alcohol as a risk factor (Table I).

### Tobacco use

The percentage of participants who have never previously smoked prior to our study was at 89.7 percent. On a positive note, only 2.2 percent of respondents reported being current smokers while 7 percent had smoked in the past but stopped. Cigarettes dominated the type of tobacco use at 77.8 percent, pipes 11.1 percent, whilst rolled and chewing took up to 5.6 percent each. Four respondents (2 percent) reported chewing some form of tobacco. Respondents smoked between 1 and 10 cigarettes a day with those reporting smokingo3, 3–5 and 6–10 were equally distributed at 27.3 percent while those at over 10 were 18.2 percent.

## Discussion

Greater Kampala with a population of 5m people consists of three neighboring local districts including Kampala, Wakiso and Mukono. Large numbers of the inhabitants of these areas tend to visit Mulago hospital for free public healthcare. Every year, at the School of Public Health Dentistry, the Rotary Club of Kampala North and the National Social Security Fund offer a weeklong free dental treatment camp that is advertised widely. The researchers took advantage of this since we felt it would give us a good distribution of patients from these districts. A high number of patients invited to this survey accepted to participate (71 percent) thus limiting any bias in generalizing our findings to other patients who attended this camp. The predominance of female individuals in the survey (56.8 percent) was similar to that reported by other centers[27, 28]. This is probably because of the higher healthcare-seeking behavior of females compared to men.

Oral cancer awareness and knowledge in our population were very poor. Over 90 percent claimed never to have heard of oral cancer; however, the fact that 22 percent of the study population pointed out pelvic cancer shows poor knowledge of cancer in general, or guesswork. Our results are in line with the report from Iran[26], but markedly different from a study conducted in India[29]. The Indian level of knowledge is probably due to the high incidence of oral cancer in that population that has helped to increase awareness. Additionally, the prevalence of the disease attracts public health messages that contrast with our low incidence setting.

Smoking as a risk factor for oral cancer was pointed out by the majority of the respondents. However, alcohol was recognized by less than 50 percent as a risk factor. This was similar to what has been reported elsewhere[27, 30]. We need to look at the public health messages we send out on oral cancer since alcohol seems to be less emphasized. In fact, a study conducted among nurses also showed a very low level of recognition of alcohol intake as a risk factor for oral cancer[31] showing a clear deficiency and need to retool our public health messages. In Uganda, we may need to do more since the reported level of alcohol consumption among our population is high[32]. Interestingly, as shown in Table I, patients who had consumed alcohol pointed out tobacco use as a risk factor but not alcohol. This emphasizes the need to get the message on alcohol more widely advertised to our populace.

Over 93.5 percent of the individuals we surveyed had never had an oral cancer screening. This is despite the fact that more than 60 percent of the participants agreed that early detection of some cancers can improve the chances of successfully treating them. Although this is similar to a report from Portugal[27], it is not surprising given the low incidence of the disease in our population[6]. Thus, it is not among the priority cancers that warrant regular screening.

Like most surveys, our study was disadvantaged by the reliance on self-reports in the presence of health workers since certain habits such as smoking may be underreported. Additionally, we relied on patients who had come for treatment at a cost-free camp so it may not be as representative of the target population. In terms of belief items, some people may not feel comfortable sharing their true beliefs with health workers. Questions on the role of pastors and witchcraft may not elicit accurate information and there is no accurate way of validating these responses. These may better be answered in a qualitative study than our kind of research design[33].

The question of education about oral cancer may not be appropriate since people who come to these free camps most likely do not visit medical or oral health practitioners except when they are indisposed. Therefore, the opportunity of such education may not arise or if it did, it would not register on the patient’s mind. Another limitation relates to the level of comprehension and translational effect on Luganda. This can make the wording of certain questions, such as ones on throat cancer, hard to distinguish from laryngeal or pharyngeal cancers. The different translations can lead to erroneous responses and results. Despite validation, the level of proficiency differs among people who feel comfortable with language due to diverse backgrounds and interference from other languages.

**Figure 1.**
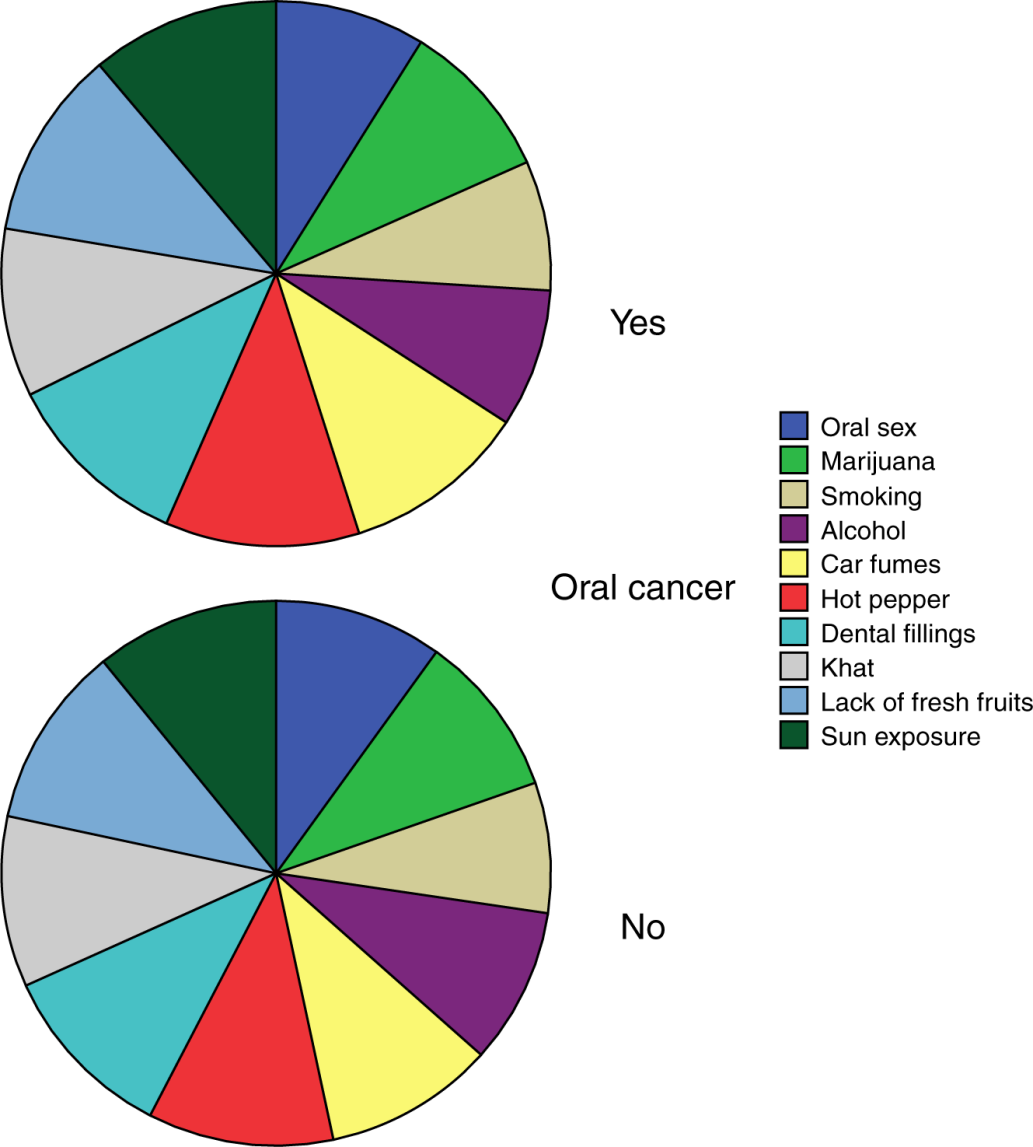
Shows responses to factors associated with oral cancer against oral cancer awareness

Our question format was for respondents to choose a response from listed options; thus this may have attenuated the lack of awareness since sometimes people will just choose unknowingly and correctly guess the right answer. Figure 1 shows the responses to what our participants knew as being linked to oral cancer. In that figure, there was no difference between those who had heard of the disease.

Although we tried to quantify the tobacco and alcohol use, the accuracy is questionable since locally made alcohol is not easily quantifiable.

## Conclusions and recommendations

There is a low level of awareness/knowledge about oral cancer in our studied population. While smoking is the highest known risk factor for oral cancer, alcohol is not recognized by more than half. Luckily, a large percentage of the population still believe in the efficacy of medical personnel in the management of oral cancer. Further, although the participants strongly agree that detection of some cancers can improve the chances of successfully treating them, the screening is very low.

Public health messages that emphasize the risk factors including alcohol use should be developed. We need to devise other methods of dissemination of these messages to reach the communities, including the use of social media and also through religious and community leaders. Further research is also recommended on a large scale in community settings to enlist bigger representation on population views on risk factors, causes and prevention of oral cancer.

### Study limitations

Our study did not carry out an item–objective congruence on the questionnaire; therefore, some items may not have captured the intended information. We also relied on consensus at arriving at the best translation and did not re-test the agreed upon version, thus creating a possibility that the English and Luganda version may not have captured the same information.

## Figures and Tables

**Table I.** The social demographic factors against the knowledge of risk factors for oral cancer

Variables	*n* (%)	Oral cancer awareness	Tobacco as a risk factor	Alcohol as a risk factor	Oral sex as a risk factor	Age < 50	Living with HIV
*Gender*							
Male	80 (42.5)	25 (31.2)	57 (71.3)	37 (46.3)	27 (33.8)	17 (21.3)	25 (31.2)
Female	108 (57.4)	34 (31.5)	68 (63.0)	48 (44.4)	37 (34.3)	26 (24.1)	44 (40.7)
*p*		0.973	0.234	0.806	0.942	0.649	0.182
*Age (years)*							
≽49	156 (83.0)	51 (32.7)	101 (64.7)	70 (44.9)	53 (34.0)	32 (20.5)	51 (32.7)
<49	31 (16.5)	8 (25.8)	23 (74.2)	15 (48.4)	10 (32.3)	11 (35.5)	18 (58.1)
Not specified	1 (0.5)	0 (0.0)	1 (100.0)	0 (0.0)	1 (100.0)	0 (0.0)	0 (0.0)
*p*		0.598	0.462	0.619	0.371	0.167	*0.021*
*Income group (monthly in $)*							
100 and below	36 (19.1)	5 (13.9)	25 (69.4)	17 (47.2)	9 (25.0)	5 (13.9)	9 (25.0)
101–249	38 (20.1)	13 (34.2)	23 (60.5)	14 (36.8)	17 (44.7)	9 (23.7)	18 (47.4)
250 plus	11 (5.6)	5 (44.5)	10 (90.9)	7 (63.6)	6 (54.5)	3 (27.3)	4 (36.4)
*p*		*0.049*	0.159	0.267	0.100	0.466	0.136
*Marital status*							
Single	119 (63.3)	40 (33.6)	81 (68.1)	54 (45.4)	45 (37.8)	22 (18.5)	44 (37.0)
Cohabit	6 (3.2)	3 (50.0)	4 (66.6)	4 (66.6)	1 (16.6)	2 (33.3)	2 (18.5)
Married	58 (30.9)	13 (22.4)	36 (62.1)	24 (41.4)	17 (29.3)	16 (27.6)	20 (34.5)
Divorced	4 (2.1)	2 (50.0)	3 (75.0)	3 (75.0)	0 (0.0)	3 (75.0)	3 (75.0)
Private	1 (0.5)	1 (100.0)	1 (100.0)	0 (0.0)	1 (100.0)	0 (100.0)	0 (100.0)
*p*		0.182	0.865	0.445	0.18	0.065	0.515
*Tobacco use*							
Ever	19 (10.1)	4 (21.1)	14 (73.8)	10 (52.6)	9 (47.4)	3 (15.8)	6 (31.6)
Never	169 (89.9)	55 (32.5)	111 (65.7)	75 (44.4)	55 (32.5)	40 (23.7)	63 (37.3)
*p*		0.306	0.483	0.493	0.196	0.438	0.625
*Alcohol use*							
Ever	82 (43.6)	27 (33.9)	62 (75.6)	42 (51.2)	30 (36.6)	25 (30.4)	34 (41.5)
Never	106 (56.4)	32 (30.1)	63 (59.4)	43 (40.6)	34 (32.1)	18 (17.0)	35 (33.0)
*p*		0.688	*0.020*	0.146	0.518	*0.029*	0.234
*Relative who died from cancer*							
Yes	62 (33.0)	20 (32.2)	48 (77.4)	27 (43.5)	26 (41.9)	17 (27.4)	29 (46.8)
No	92 (48.9)	29 (31.5)	60 (65.2)	43 (46.7)	28 (30.4)	21 (22.8)	34 (37.0)
Not sure	34 (18.1)	10 (29.4)	17 (40.0)	15 (44.1)	10 (29.4)	5 (14.7)	6 (17.6)
*p*		0.959	*0.023*	0.917	0.276	0.366	*0.018*

Note: Values in italics are significant at p = 0.05

**Table II.** Risk factors and determinants of the well-being of cancer patients on a Likert scale

*n* (%)	Strongly agree	Agree	Somewhat agree	Disagree	Strongly disagree	Not sure
Early detection of some cancers can improve the chances of successfully treating them	117 (62.2)	35 (18.9)	3 (1.6)	5 (2.7)	2 (1.1)	25 (13.5)
Who develops cancer and who does not is a matter of chance, so there is nothing anybody can do to avoid it	48 (25.4)	40 (21.1)	14 (7.6)	33 (17.8)	16 (8.6)	37 (19.5)
Some people can make changes in the way they live to reduce their risk of developing cancer	99 (52.4)	46 (24.3)	8 (4.3)	4 (2.2)	4 (2.2)	27 (14.1)
Witchcraft can lead to the development of cancer	12 (6.5)	14 (7.6)	9 (4.9)	52 (27.6)	45 (23.8)	56 (29.7)
